# An Exploratory Study of Physiological Linkage Among Strangers

**DOI:** 10.3389/fnrgo.2021.751354

**Published:** 2022-02-07

**Authors:** Savannah M. Boyd, Ashley Kuelz, Elizabeth Page-Gould, Emily A. Butler, Chad Danyluck

**Affiliations:** ^1^Department of Psychology, University of Arizona, Tucson, AZ, United States; ^2^Department of Family Studies and Human Development, University of Arizona, Tucson, AZ, United States; ^3^Department of Psychology, University of Toronto, Toronto, ON, Canada; ^4^Department of Psychology, Carleton University, Ottawa, ON, Canada

**Keywords:** physiological linkage, strangers, *rties* package, context, affiliation

## Abstract

The present study explores physiological linkage (i.e., any form of statistical interdependence between the physiological signals of interacting partners; PL) using data from 65 same-sex, same ethnicity stranger dyads. Participants completed a knot-tying task with either a cooperative or competitive framing while either talking or remaining silent. Autonomic nervous system activity was measured continuously by electrocardiograph for both individuals during the interaction. Using a recently developed R statistical package (i.e., *rties*), we modeled different oscillatory patterns of coordination between partner's interbeat interval (i.e., the time between consecutive heart beats) over the course of the task. Three patterns of PL emerged, characterized by differences in frequency of oscillation, phase, and damping or amplification. To address gaps in the literature, we explored (a) PL patterns as predictors of affiliation and (b) the interaction between individual differences and experimental condition as predictors of PL patterns. In contrast to prior analyses using this dataset for PL operationalized as covariation, the present analyses showed that oscillatory PL patterns did not predict affiliation, but the interaction of individual differences and condition differentially predicted PL patterns. This study represents a next step toward understanding the roles of individual differences, context, and PL among strangers.

## Introduction

The need to establish and maintain interpersonal relationships is a primary human motivation (Baumeister and Leary, 1995), to the point that the maintenance of stable relationships is crucial for survival (Maslow, 1943; Bowlby, 1969). Co-construction of affiliative bonds necessitates attunement to complex emotional states between both members of a pair. Subcomponents of emotion, including experience, behavior, and physiology, interact within and between people, with a greater likelihood of mutual influence when these processes occur between individuals in already established relationships (Butler, 2011). However, given that all relationships begin with two strangers, what is less clear is how these subcomponents interact to produce the state of affiliation that is indispensable for relationship building. Focusing on the physiological domain, physiological linkage (PL) refers to any form of statistical interdependence between the physiological markers of two or more people over time and could provide the underlying support for affiliative processes (Butler, 2011).

There are several strengths for utilizing PL to investigate interpersonal processes such as affiliation. First, PL operates unconsciously and automatically, which offers a more sensitive and objective estimate of interpersonal processes than self-report measures (Butler and Randall, 2013). Second, PL offers high temporal resolution, given that the signals can be measured in second-by-second intervals (Reed et al., 2013). Third, there is mounting evidence that autonomic nervous system activity (ANS) is associated with both intra- and interpersonal processes, including emotion regulation and prosocial behavior (Ekman et al., 1983; Levenson and Ruef, 1992; Porges, 2003; Grossman and Taylor, 2007; Kreibig, 2010).

The study of PL emerged in the 1950s as a method of measuring therapeutic rapport (e.g., Di Mascio et al., 1955; Coleman et al., 1956). Since then, PL has been extensively examined in parent-child, therapist-client, friend, romantic dyads, and strangers (see Timmons et al., 2015; Palumbo et al., 2017 for systematic reviews). In line with the present study's focus on strangers, two studies have examined PL among stranger dyads who were conversing and found significant electrodermal activity linkage between partners who were matched on gender (Guastello et al., 2006) and those paired with an opposite-gender partner (Silver and Parente, 2004). Other studies have measured PL between co-present strangers watching movies but not interacting (Golland et al., 2019) or working as teammates toward a shared goal (Henning et al., 2001; Elkins et al., 2009; Behrens et al., 2020). These studies showed that (1) PL can be driven by a shared environment and (2) PL can predict team performance. In addition, studies have found that both cooperative and competitive conditions give rise to PL among stranger dyads, which highlights the importance of context when interpreting PL (Chanel et al., 2012; Strang et al., 2014; Vanutelli et al., 2017, 2018). In a recent effort to understand the interactive influence of such contexts, Danyluck and Page-Gould (2019) found that parasympathetic covariation predicted affiliation. However, this association was moderated by social context such that affiliation was lower in competitive contexts compared to cooperative contexts.

The studies that have examined PL among strangers share some limitations with many studies examining PL in other types of dyads. First, studies investigating PL have used methods that neglect the dynamic nature of physiological signals. For example, cross-correlational methods do not capture signal damping (i.e., movement toward baseline) or amplification (i.e., movement away from baseline), both of which could be related to the degree of environmental engagement or coregulation (i.e., the biological, psychological, or behavioral interdependence of partners that supports change and stability) (Butler and Randall, 2013; Reed et al., 2015; Li X. et al., 2020). Thus, a combination of statistical methodology and contextual factors could explain why PL correlates with diametrically opposed outcomes across studies, including associations with both prosocial (e.g., relationship satisfaction, affiliation, and better team performance) and antisocial behaviors (e.g., relationship conflict and dissatisfaction). Second, there is a potential for a reciprocal relationship between PL and people's perceptions of their partner. Specifically, perceptions of one's partner could change as a function of PL experienced during an interaction. However, simultaneously perceptions of one's partner may be related to how one approaches and adapts to various difficulties and transitions during the interaction, resulting in different PL patterns (Butler, 2011). For example, one may start to view a partner more negatively during an interaction in which PL is contributing to amplified physiological stress responding, but at the same time, the negative views could contribute to stressful PL. In other words, PL can determine or be determined by the quality of the interaction. Third, people bring a host of pre-existing traits to social interactions. Drawing from the adult attachment literature, individuals have beliefs about themselves and others that influence all aspects of their social interactions. For instance, attachment styles impact the experience, encoding, retrieval, and manipulation of affective information as well as the motivation to approach social interactions (see Schwartz et al., 2007; Shaver and Mikulincer, 2007; Ravitz et al., 2010 for a review). In addition, dispositional levels of social traits may influence (a) how one behaves during an interaction and (b) how one rates the interaction after it ends. Furthermore, individuals in a stranger dyad may present with mismatched levels of specific traits, leading to different interaction dynamics. In summary, although it has not typically been considered, there is ample reason to think that we will need to consider individual differences to understand PL fully.

The present study addresses these limitations by using secondary data from a project that examined PL among same-sex, same-ethnicity strangers who participated in a between-subjects 2 (Social interaction: Talking vs. No Talking) by 2 (Interaction orientation: Competition vs. Cooperation) experiment (Danyluck and Page-Gould, 2019). To model the physiological data, we used the new *rties* package, available within the statistical computing platform R. The *rties* package simplifies modeling of both individual and dyad level physiological dynamics during social interactions and can represent nuanced patterns, or trajectories, of PL based on frequency, coupling, amplification, and damping of the signals over time (Butler and Barnard, 2019). We use these dynamic patterns as predictors of affiliation (i.e., liking and similarity) and as outcomes of individual differences related to social behavior (i.e., attachment, social anhedonia, and social skills). Therefore, this exploratory study focuses on the following research questions:

(1) Do distinct PL patterns emerge across dyads?
(2) Do PL patterns predict affiliation?
(3) Do PL patterns predict affiliation differently across conditions?
(4) Are PL patterns predicted by condition?
(5) Are PL patterns predicted by individual differences or affiliation?
(6) Are PL patterns predicted by individual differences or affiliation differently across conditions?

## Background and Empirical Studies

### Introducing Physiological Linkage

Most investigations of PL use signals from the autonomic nervous system (ANS; Palumbo et al., 2017). The ANS is comprised of the sympathetic nervous system (SNS) and the parasympathetic nervous system (PNS). The two branches work together to regulate organ functioning in the cardiac, respiratory, and endocrine systems. Classically, the SNS is called “the fight or flight” system, which assists in arousal and energy expenditure that ready the body for action. The PNS, colloquially called the “rest and digest” system, aids in decreased arousal, energy storage, and recuperation (Levenson et al., 2016). The SNS and PNS work together to balance inputs based on environmental demands (Porges, 2003). Physiological signals from the ANS can be distinguished by the degree of SNS and PNS influence. For example, in the present research we focus on heart rate, or more specifically the interbeat interval (IBI)—the time interval between heartbeats—because it reflects the joint action of the SNS and PNS on the heart, making it a sensitive indicator of physiological dynamics during social interaction (Levenson et al., 2016).

Current research on PL focuses on determining whether statistical interdependence of one sort or another (e.g., cross-correlations and between-partner regression betas) arises between people for an autonomic marker (e.g., respiratory sinus arrhythmia, skin conductance, or IBIs), and to what extent other variables of interest (e.g., relationship satisfaction) are associated with this interdependence when it exists (see Palumbo et al., 2017 for a review). Interestingly, PL is associated with a range of seemingly contradictory outcomes. While PL is associated with prosocial behaviors like affiliation (Danyluck and Page-Gould, 2018, 2019), PL is also associated with relationship dissatisfaction (Levenson and Gottman, 1985) and heightened inflammatory response among couples in conflict (Wilson et al., 2018). Therefore, it is naïve to assume that PL is an all-or-none process predictive of only positive or negative outcomes. Instead, consideration of quantitative and qualitative characteristics of the physiological signals themselves, as well as the context(s) in which they are occurring, is warranted.

Different PL patterns can be characterized by (a) *frequency of oscillation* (i.e., number of oscillations per unit in time), (b) *relative phase* (i.e., whether partner's are rising and falling in-phase or anti-phase with each other), and (c) *damping and amplification* (i.e., movement toward or away from baseline). Considering these characteristics in more detail, first, physiological signals (e.g., heart rate and respiration) show oscillations arising due to the complex interplay between external perturbations and internal homeostatic processes (Helm et al., 2012; Reed et al., 2015). Different PL patterns can show a range of frequencies, from high frequency (rapid oscillations) to low frequency (slow oscillations). Second, the partners' physiology can be either in-phase (e.g., when one partner is high, so is the other) or anti-phase (e.g., when one partner is high and the other is low). Third, physiological signals can show damping and/or amplification over time, with feedback loops contributing to the stability of the signal. Negative feedback loops (i.e., A leads to B, and subsequently B inhibits A) serve to damp physiological oscillations and stabilize the signal over time. In contrast, positive feedback loops (i.e., A leads to B, and subsequently B produces more of A) amplify physiological oscillations and destabilize the signal over time. Lastly, as implied from the definition of PL, dyads' physiological signals can covary and become coupled over time. Coupling refers to the degree of coordination of the partners' physiological signals over time (Steele and Ferrer, 2011; Helm et al., 2012; Reed et al., 2015). Based on the prior three characteristics and degree of coupling, many types of possible PL patterns can arise (Reed et al., 2013). For example, partners' physiology can be changing in the opposite direction but amplify together over time, or partners' physiology can be changing in the same direction but damp together over time (Reed et al., 2013). As another example, a recent study by Li X. et al. (2020), using the *rties* package to model PL among same-sex male couples, found two PL patterns characterized by differences in frequency, phase, and damping. In summary, to understand the precursors and consequences of PL, we need to consider the variety of patterns that can emerge.

### Physiological Linkage Among Strangers

Empirical studies investigating PL among strangers have consistently shown that strangers' physiology can become linked (Palumbo et al., 2017). Again, emphasizing the importance of context and type of interaction, some studies have found linkage of cardiac markers even among strangers who did not interact with each other but shared only the same environment (Golland et al., 2019; Behrens et al., 2020; Bizzego et al., 2020). Other studies have investigated PL among strangers under more naturalistic conditions. For example, significant covariation of electrodermal activity has been shown among strangers who were engaging in an unstructured conversation (Silver and Parente, 2004; Guastello et al., 2006). Similarly, Scarpa et al. (2018) detected PL, as measured by IBIs, among strangers who participated in a turn-taking conversation about emotional topics. In the gaming and teaming literature, PL has been examined in newly constructed teams consisting of strangers. For instance, PL of cardiac and electrodermal markers has been seen among teammates working toward a common goal (Henning et al., 2001; Elkins et al., 2009; Strang et al., 2014). Combining the effects of context and naturalistic conversation, a recent study by Danyluck and Page-Gould (2019), using the same data set as the present report, investigated PL among strangers by manipulating context (i.e., competitive or cooperative) and interaction orientation (i.e., talking or not talking). Results showed that a simple indicator of PL, as measured by cross-correlation, emerged across contexts in both the sympathetic and parasympathetic branches of the ANS. Although these studies provide supporting evidence of PL among strangers, the present work extends them by considering complex patterns of PL as both predictors and outcomes of individual differences and affiliation.

### Social Context

More than 40 studies have assessed PL across a range of cooperative and competitive or conflict-laden contexts and within a range of relationship types, including romantic partners (Levenson, 1983; Helm et al., 2014; Chen et al., 2020), parents and their children (Giuliano et al., 2015; McKillop and Connell, 2018; Li Z. et al., 2020), friends (Chanel et al., 2012; Järvelä et al., 2014), acquaintances (Codrons et al., 2014), and strangers (Kraus and Mendes, 2014; Danyluck and Page-Gould, 2018, 2019). By and large, PL has been ubiquitous throughout each of these studies. Nevertheless, few have manipulated cooperation and competition within the same experiment and, thus, it remains unclear whether one context is especially likely to elicit PL than the other. Eight studies have contrasted PL across both cooperative and competitive contexts, and all but one included members of pre-existing relationships (e.g., friends, romantic couples, or classmates). Moreover, findings are mixed across these studies. In some studies, PL magnitude was greater during cooperation than during conflict (e.g., Woltering et al., 2015) but the reverse was true in other studies (e.g., Järvelä et al., 2014). In some cases, there was no distinction in PL across cooperative or competitive contexts (e.g., Helm et al., 2014; Danyluck and Page-Gould, 2019). Moreover, within one study, whether cooperation or competition predicted greater magnitude PL depended on the physiological measure (e.g., IBI and respiration vs. high frequency heart rate variability; Chanel et al., 2012). Thus, it remains unclear whether cooperation or competition/conflict are more or less likely to elicit PL and this is especially true of PL between strangers, where research is particularly limited. Given the importance of initial social interactions for the development of friendships and the role that PL might play in fostering such relationships (Danyluck and Page-Gould, 2018, 2019; Page-Gould et al., in press), we view it as particularly important to examine whether and to what extent variation in social contexts affects PL and affiliative processes between strangers.

### Affiliation

Affiliation is defined as an individual's desire to “establish, maintain, or restore” relationships with others or groups (Heyns et al., 1958). Murray (1938) proposed that seeking friendly association with others who resemble one, like one, or whom one likes is a fundamental human desire. The affiliative process is primarily based on liking or personal attachment and constitutes the first step of relationship building (Hofer and Hagemeyer, 2018). Notably, individuals make affiliative judgments extremely quickly. For example, individuals decide what type of relationship to pursue (e.g., friendship or acquaintance) in the first minutes of an initial encounter (Berg and Clark, 1986). Reciprocal liking, mutual self-disclosure, and similarity are significant determinants of interpersonal attraction across dyadic relationship types (i.e., romantic relationships, same, and opposite-sex friendships; Campbell et al., 2015). Despite the importance of affiliation, little is known about whether PL patterns contribute to or arise from affiliation, so we address this question in the present study.

### Individual Differences

Beliefs about self and the fear of rejection can hinder the formation of affiliative bonds (Bowlby, 1969; Schwartz et al., 2007; Hofer and Hagemeyer, 2018). For example, attachment styles differentially predict motivations for affiliation. More specifically, anxious attachment has been positively associated with attention-seeking, and avoidant attachment has been negatively associated with emotional support (Schwartz et al., 2007). In already established relationships, anxiety can be beneficial, to a certain extent, by serving as a signal to repair relationships. However, interactions with strangers are a “gamble” (Brosnan et al., 2017). On the one hand, one can unlock new resources and support by affiliating with strangers. On the other hand, interactions with strangers involve ongoing judgments about when the environment is safe to share and make individuals vulnerable to rejection. Therefore, it is essential to consider individual factors that could impact the affiliative process among strangers.

Based on a review of the PL literature more broadly, PL may represent psychosocial variables that operate at the physiological level (Palumbo et al., 2017), which necessitates the examination of individual differences that may interact with environmental conditions to produce PL. Given the exploratory nature of this study, we focused on variables that could (a) reflect individual-level dispositions, (b) shape perceptions of affiliation, and/or (c) influence physiological responding. Several variables cut across these domains, including *attachment* (for a review Schwartz et al., 2007; Ravitz et al., 2010; Schreiber et al., 2021), *social anhedonia* (i.e., the reduced ability to experience pleasure from social experiences; Meehl, 1962; Llerena et al., 2012), *social skills* (i.e., verbal and non-verbal behaviors necessary for initiation of the affiliative process; Walker et al., 1995; Blanchard et al., 2015), and *social anxiousness* (Leary, 1983; Kashdan and Roberts, 2004).

### Exploratory Hypotheses

Given the dearth of previous studies in this area, we do not offer *a priori* hypotheses. However, based on a recent study by Li X. et al. (2020) that utilized the same modeling approach, we expected that: (1) at least two qualitatively distinct patterns of PL would emerge, (2) the patterns would differentially predict affiliation, perhaps moderated by condition, and (3) the patterns would be predicted by condition, affiliation, and individual differences, perhaps moderated by condition.

## Materials and Methods

### Participants

This dataset comes from a more extensive study examining intragroup interactions at the University of Toronto, where introductory psychology students and community members completed a 2-h study. Study procedures and materials were approved by the University of Toronto Institutional Review Board. Participants included in this study provided written consent prior to engaging in study procedures. Participants were paired with a same-sex, same-ethnicity partner before coming in to complete the experiment, given that the original goal of data collection was to examine intragroup interactions. The original sample consisted of 68 dyads (*N* = 136). One cross-sex dyad was accidentally scheduled, and two dyads had partial physiological data. Thus, the final sample consisted of 65 dyads (*N* = 130), was ~70% female and the mean age was 20.5 years (*SD*_*age*_ = 7.43). The sample was relatively diverse: 40% East Asian, 32% White, 11% South Asian, 5% Southeast Asian, 4% Middle Eastern, 4% Black, 3% West Indian, 1% multi-ethnic, and 1% Hispanic. Additionally, 62% of the sample reported making < $5,000 per year. Regarding compensation, participants had the option to receive course credit, $20.00, or a combination of course credit and money.

### Experimental Procedure

Participant pairs were randomly assigned to one of four conditions based on a 2 (Social Interaction, between-subjects: Talking vs. No Talking) × 2 (Interaction Orientation, between-subjects: Cooperative vs. Competitive) between-dyad design. After providing consent and completing pre-task questionnaires, participants were connected to the physiological recording equipment. A physiological baseline recording was collected for 5 min before beginning the task. After the baseline recording, participants were instructed to complete a knot-tying task using strings attached to their chairs. Each participant received a three-foot string and were instructed to use their dominant hand to tie as many knots as possible during a 5-min period. The degree of sociality of the task was manipulated: Participants were assigned to get to know each other while completing the task (Talking Condition) or to remain silent (No Talking Condition). The type of social interaction was manipulated: Participants were told they would receive points to win an Amazon gift card for each knot that the dyad tied collectively (Cooperative Condition) or for each knot more that they tied relative to their partner (Competitive Condition). Based on the condition, research assistants read a different framing script before starting the task. The scripts are described in detail below.

**Talking-cooperative**. Pairs in the talking-cooperative condition were given the following instructions:
  We want you to get to know your partner while working toward a collaborative goal. You will both receive one long string in your dominant hand. We want you to tie as many knots as possible in 5 min on one long string using one hand. This is a fun party-game, designed to encourage social affiliation and cooperation. You should be trying to get to know each other at the same time as completing this task so feel free to ask each other personal questions and at the same time try to cooperate with each other on this task. The more knots you can tie as a team, the more points you will each receive. The team with the highest points will be entered into a draw to receive two $50.00 gift cards at Amazon.ca.
**Talking-competitive**. Pairs in the talking-competitive condition were given the following instructions:
  We want you to get to know your partner while competing for points/rewards. You will both receive one long string in your dominant hand. We want you to tie as many knots as possible in 5 min on one long string using one hand. This is a fun game, designed to encourage social affiliation and competition. You are competing against each other for a small reward, but you should try to get to know each other at the same time so feel free to socialize and to ask each other personal questions. The more knots you can tie as an individual, the more points you will receive. Whoever has the most points will be entered into a draw to receive a $50.00 gift card at Amazon.ca.
**No talking-cooperative**. Pairs in the no talking-cooperative condition were given the same instructions as the talking-cooperative participants, but they were instructed not to talk: Do not socialize, do not talk. Just work on the task. The more knots you can tie as a team, the more points you will each receive.
**No talking-competitive**. Pairs in the no talking-competitive condition were given the same instructions as the talking-competitive condition, but they were instructed not to talk to each other: Do not socialize, do not talk. Just work on the task. You are competing against each other.

### Measures

#### Interbeat Interval

Interbeat interval (IBI) refers to the time between subsequent R waves or the time to complete one cardiac cycle in milliseconds. This measure results from the coordinated activity of both sympathetic and parasympathetic branches of the ANS. Given that IBI changes within a few seconds, it affords high temporal precision for PL. Cardiovascular activity was continuously measured by electrocardiogram (ECG) for all participants throughout the interaction. ECG was recorded with electrodes in the modified Lead II placement and sent to a computer *via* Biopac ECG100C Module and MP150 amplifier (Biopac Systems, Inc., Goleta, CA). The ECG data were scored with Acq*Knowledge* version 4.4 (Biopac Systems, Inc., Goleta, California) to extract IBIs. The IBI data was segmented into 2-s intervals. Next, the mean over each 2-s interval was calculated for the entire 5-min conversation, resulting in 150 observations for each participant.

#### Pre-task Measures

##### Attachment

We used the 27-item *Attachment Style Questionnaire* (ASQ; Feeney et al., 1994). Each item was rated using a 6-point Likert-type agreement scale ranging from 1 (*totally disagree*) to 6 (*totally agree*). The short-form scale consists of three subscales: secure, anxious, and avoidant attachment. The 6-item secure attachment subscale reflects one's confidence in establishing relationships with others (e.g., “I feel confident about relating to others”; *M* = 3.83, *SD* = 0.73; α = 0.74). The 9-item anxious attachment subscale measures one's attention-seeking in and pre-occupation about relationships (e.g., “I worry a lot about my relationships”; *M* = 3.46, *SD* = 0.92, α = 0.86). The avoidant attachment subscale consists of 12 items that measure one's unease with closeness in relationships and reluctance to trust others (e.g., “I prefer to keep to myself”; *M* = 3.10, *SD* = 0.59, α = 0.75). Items were reverse coded such that higher scores indicated higher levels of each type of attachment.

##### Interaction Anxiousness

Interaction anxiousness was measured with a 9-item questionnaire consisting of items relating to one's level of anxiety in different social situations (Leary, 1983; e.g., “I feel anxious in social situations”; “I often feel nervous even in casual get togethers”). Each item was rated on a 5-point Likert-type scale (1 = *not at all* to 5 = *extremely*). A mean score was calculated for each person (*M* = 2.35, *SD* = 0.79). This scale had sufficient internal reliability (α = 0.87).

##### Social Anhedonia

We used the 15-item Revised Social Anhedonia Scale from the Wisconsin Schizotypy Scales (Winterstein et al., 2011) to measure one's inability to experience pleasure from social interactions (e.g., “Making new friends isn't worth the energy it takes”; *M* = 1.81, *SD* = 0.78, α = 0.86). Each item was rated on a 7-point scale ranging from 1 (strongly disagree) to 7 (strongly agree). Items were reverse-scored such that higher scores indicated high levels of social anhedonia.

##### Social Skills

We used a 5-item face-valid scale to measure the perception of one's social skills before the interaction (e.g., “In the upcoming interaction, I see myself as making a good first impression”; *M* = 5.11, *SD* = 1.02, α = 0.91) and perception of one's partner's social skills (e.g., “In the upcoming interaction, I see my partner as being at ease”; *M* = 4.98, *SD* = 0.95, α = 0.95). Each item was rated on a 7-point scale ranging from 1 (*not resourceful at all*) to 7 (*extremely resourceful*).

#### Post-task Measures

##### Affiliation

Perceived affiliation with one's partner was measured with a 24-item questionnaire (e.g., “How much do you like your partner?”; “How sociable was your partner during the interaction?”) that was rated on a 7-point scale (1 = *not at all* to 7 = *very*). Given that this measure was developed for this study by pulling items from various sources, the internal structure was unknown. To explore whether this scale assessed one underlying construct or multiple ones, an exploratory factor analysis (EFA) was completed. After omitting the items that loaded on multiple factors, three distinct factors emerged. The factors are described briefly here. Factor 1 (i.e., similarity to partner and likelihood of future friendship) was comprised of 6 items (α = 0.94). Factor 2 (i.e., positive personal qualities of one's partner, including genuine and helpful) was comprised of 5 items (α = 0.83). Factor 3 (i.e., poise and comfort during the interaction) was comprised of 2 items (α = 0.73). Each subscale was used as an outcome or predictor for the research questions referencing affiliation.

##### Similarity

Perceived similarity to partner was measured after the task using a 5-item face-valid questionnaire created for this study (e.g., “My partner and I are very similar”; “My partner and I share a lot in common”) that was rated on a 7-point scale (1 = *not at all* to 7 = *very*). The mean was 4.45 (*SD* = 0.99) and the alpha was 0.92.

### Analytic Approach

We conducted analyses using the R Statistical Platform version 3.6.3 (R Core Team, 2020) using the following two steps:

#### Step 1: Modeling Physiological Linkage

In the present study, same-sex, same-ethnicity dyads completed a knot tying task in a between-dyad 2 (Social interaction: Talking vs. No Talking) by 2 (Interaction orientation: Competition vs. Cooperation) design. Therefore, each dyad participated in only one of four possible conditions. To model IBI linkage throughout the task for each dyad, we used the *rties* package v5.0.0 (Butler and Barnard, 2019) to estimate a coupled oscillator (CO) model. For more details, see the vignettes associated with the *rties* package. Briefly, the *rties* coupled oscillator (CO) model is implemented as a two-intercept dyadic multilevel model that predicts the second-derivative of the observed variable (IBI in this study) for each partner from four parameters for each partner: (a) each person's own IBI time series, which is related to frequency of oscillations, (b) the first derivative of each person's IBI time series, which indicates damping/amplification, (c) each person's partner's IBI time series, which indicates coupling in regard to frequency, and (d) each person's partner's first derivative of their IBI time series, which indicates coupling regarding damping/amplification. Of note, the *rties* package uses an idiographic modeling approach whereby the CO model is applied to each dyad one at a time. Therefore, a total of eight parameters were generated for each dyad and written into a separate data frame that is used in the next step of the analysis. Given that the CO model requires individual-level distinguishable data, we created an arbitrary distinguishing variable (i.e., 0 vs. 1) for the dyads as they were indistinguishable based on sex or ethnicity. In this context, the arbitrary distinguishing variable serves a data-organization function and does not enter the analyses.

Using the tools provided by *rties*, all data were linearly detrended and the intercept was removed (Boker and Laurenceau, 2006) by estimating the residuals from the person's IBI as predicted from time. To begin building the CO model, the first and second derivatives of the observed variable are estimated from the data using Local Linear Approximation (Boker and Nesselroade, 2002). To generate the derivatives, three sets of parameters are specified by the user: delta, tau, and embed. See the *rties* vignettes for an explanation of these parameters and steps for choosing values. Results will vary depending on the choice of these values and so care is required at this step. We considered a range of possible values and chose the combination for each dyad that maximized the *R*^2^ for the CO model fit to their data (Boker and Nesselroade, 2002). In the present analyses, delta was set to 1, tau could be 3 or 4 and embed could be 3, 4, or 5. The output details the combinations of tau and embed values that produced the maximal *R*^2^ for each dyad. This information can be used to determine model fit for each dyad and across dyads. Additionally, the mean oscillation period of the physiological data is returned, which is important for establishing that the signal was oscillating within the time period of the typical conversation. For example, if a mean oscillation of 6 min was found, but the conversations were only 5 min, then an oscillatory model would not be appropriate.

For the next step of modeling in *rties*, the set of 8 parameter estimates for each dyad can be used as indicators in a Latent Profile Analysis (LPA) that groups dyads based on dynamics throughout the task. Because the CO model represents non-linear dynamics, the behavior of the dyadic system cannot be intuited by examining individual parameter estimates from the model. Rather, they operate together as a set to produce potentially complex temporal trajectories of both partner's observed physiological variables. Thus, when it comes to using the dynamic parameter estimates to predict or be predicted by variables, such as experimental context, we wish the parameters to be operating as a set, not as isolated individual predictors or outcomes (as would be true if used as predictors in a regression model). Instead, we use them as input to a latent profile analysis to estimate qualitatively distinct groups of dyads based on the dynamics assessed by the CO model. LPA is a type of Gaussian Mixture Model, which assumes an underlying multivariate normal distribution and groups cases based on a set of observed variables (in our case the CO parameter estimates for each dyad). In much of the literature on LPA, the focus is on trying to estimate the “true” number of underlying profiles by using fit statistics such as the BIC, but simulation studies show that this process does not work very well unless one has a huge sample and large effect size (see for example Tein et al., 2013). Thus, we did not consider fit statistics for our analyses, but instead chose the number of profiles based on: (1) interpretability in terms of meaningful dynamics, (2) the number of cases assigned to each profile, and (3) how well-separated the profiles were.

Table 1 displays descriptive statistics for the CO model, including the adjusted overall *R*^2^-value and the period of oscillation. The adjusted *R*^2^ ranged from 0.36 to 0.89, showing that the CO model fit the data well for all dyads. Given that IBIs were segmented into 2-s units, the length of the average oscillation period was about 48 s for both persons (i.e., 24.1 units ^*^ 2 s/60 min = 0.8 min or 48 s), which allows for about 6 oscillations per conversation.

**Table 1:** Descriptive statistics for the CO model.

	**MEAN**	**SD**	**MIN**	**MAX**
*R* ^2^	0.57	0.10	0.36	0.89
Period (person A)	24.88	11.18	11.67	52.53
Period (person B)	24.10	9.67	12.11	52.26

#### Step 2: Models and Testing

Step 2 involved specifying models to evaluate the exploratory aims of the study. For research questions in which PL Profile was the predictor, we included a random intercept to account for the nesting of a person in a dyad. For models containing individual-level variables, we assessed both between-dyad variation (i.e., the average of the two persons' reports) and within-dyad variation (i.e., the discrepancy between the persons' reports). Given that it is unlikely that two strangers will present with the same levels on each of these variables, these types of average difference models (Kenny, 1996) can be used to fully account for both persons' reports and understand whether within- or between-dyad variation may be responsible for observed effects. See Table 2 for details.

**Table 2:** Models for each research question.

**Research question**	**Predictor**	**Model**
Do PL patterns predict affiliation?	PL profile	Affiliation _person i, dyad j_ = π_0j_ + profile Similarity _person i, dyad j_ = π_0j_ + profile Factor 1 (similarity) _person i, dyad j_ = π_0j_ + profile Factor 2 (positive qualities) _person i, dyad j_ = π_0j_ + profile Factor 3 (comfort) _person i, dyad j_ = π_0j_ + profile
Do PL patterns predict affiliation differently across conditions?	PL profile	Affiliation _person i, dyad j_ = π_0j_ + profile × condition Similarity _person i, dyad j_ = π_0j_ + profile × condition Factor 1 (similarity) _person i, dyad j_ = π_0j_ + profile × condition Factor 2 (positive qualities) _person i, dyad j_ = π_0j_ + profile × condition Factor 3 (comfort) _person i, dyad j_ = π_0j_ + profile × condition
Are PL patterns predicted by individual differences or affiliation?	Affiliation, similarity, factors 1–3, interaction anxiousness, attachment, social anhedonia, social skills	Profile = Dyad average on *predictor* + dyad difference on predictor^*^
Are PL patterns predicted by individual differences or affiliation differently across conditions?	Affiliation, similarity, factors 1–3, interaction anxiousness, attachment, social anhedonia, social skills	Profile = Dyad average on *predictor* × condition + dyad difference on *predictor* × condition^*^

* *Ran this model for each predictor separately*.

Each model was estimated using *brms* 2.11.5, an *R* statistical package that pairs with Stan to estimate Bayesian models (Bürkner, 2017b). We favored Bayesian modeling as an alternative to the Null-Hypothesis Significance Test (NHST) for statistical inference. Given that NHST confidence intervals (CIs) imply that if one was to sample from the population 100 times and generate 100 95% CIs, 95 of those CIs would contain the population mean and five would not; there is no way to know whether the interval from a particular sample contained the population mean or not. In contrast, Bayesian estimation models the uncertainty of the parameters by generating the posterior distribution and providing the highest density interval (HDI). Put simply, the HDI allows researchers to estimate the probability that the population parameter value falls into the given range, given the data and the model. The width of the HDI reflects the degree of uncertainty of beliefs. If the HDI is wide, beliefs are uncertain (e.g., if the HDI for a predictor contains 0, it is not a likely predictor of the outcome). If the HDI is narrow, beliefs are relatively certain (e.g., if the HDI for a predictor ranges from 0.15 to 0.20, it is a likely predictor of the outcome; Kruschke and Liddell, 2018).

Given the limited availability of relevant literature, we used the *brms* default priors, which for the fixed effect parameters (e.g., those relevant to our hypotheses) are uniform distributions over the real numbers (see Bürkner, 2017a for more details about the default prior distribution). Convergence of the models was confirmed using R-hat values (i.e., should be <1.1), effective sample sizes, and visualization of trace plots. There were no convergence issues. However, the number of chains and iterations was increased (i.e., to 10 and 10,000, respectively) to stabilize the posterior distribution estimates.

## Results

### Descriptive Statistics for Self-Report Variables

Table 3 displays the descriptive statistics and Table 4 displays the Bayesian Pearson correlation coefficients for the self-report measures as calculated using the open-source software JASP (version 0.14.1; JASP Team, 2020). In JASP, we selected the Bayes Factor (BF) that corresponded to the strength of evidence for the correlation existing (i.e., BF_10_). According to the BF interpretation by Jeffreys (1961), a BF_10_ of below 3 shows anecdotal or no evidence for the hypothesis; BF between 3 and 10 shows moderate evidence; BF >10 shows strong evidence; BF >30 shows very strong evidence; BF >100 shows extreme evidence. All correlations were in the anticipated direction. For example, secure attachment negatively correlated with avoidant and anxious attachment, social anhedonia, and interaction anxiety. Furthermore, interaction anxiety positively correlated with avoidant attachment, anxious attachment, and social anhedonia. The factors measuring positive qualities of one's partner and comfort during the interaction were positively correlated with similarity and affiliation variables.

**Table 3:** Summary of self-report variables for 65 stranger dyads.

	**Mean**	**SD**	**Min**	**Max**
Age	20.94	7.43	17.00	68.00
Interaction anxiousness	2.44	0.78	0.67	4.56
Secure attachment	3.80	0.75	2.33	5.67
Avoidant attachment	3.12	0.58	1.58	4.58
Anxious attachment	3.57	0.90	1.33	5.44
Anhedonia	2.16	0.85	0.60	4.53
Social skills (self)	5.12	0.89	3.00	6.67
Social skills (partner)	4.95	0.92	3.17	7.00
Similarity	4.53	0.92	2.20	6.40
Affiliation	4.71	0.84	2.88	6.79
Factor 1 (similarity)	4.51	1.02	2.00	7.00
Factor 2 (positive qualities)	4.59	0.90	3.20	6.60
Factor 3 (comfort)	5.12	0.96	3.00	7.00

**Table 4:** Pearson correlation coefficients for self-report variables.

**Variable**	**Sex**	**Age**	**SES**	**Interaction anxiety**	**Secure att**.	**Avoidant** **att**.	**Anxious att**.	**Anhedonia**	**Social skills (self)**	**Social skills (partner)**	**Similarity**	**Affiliation**	**Factor 1 (similarity)**	**Factor 2 (positive qualities)**	**Factor 3 (comfort)**
1. Sex	—														
2. Age	0.03	—													
3. SES	−0.13	−0.08	—												
4. Interaction anxiety	−0.16	−0.26	0.15	—											
5. Secure att.	0.10	0.22	−0.31	−0.67^***^	—										
6. Avoidant att.	0.08	−0.18	0.04	0.36^*^	–0.56^***^	—									
7. Anxious att.	−0.19	−0.27	−0.16	0.48^***^	−0.51^***^	0.22	—								
8. Anhedonia	0.14	−0.17	0.23	0.38^*^	−0.63^***^	0.57^***^	0.19	—							
9. Social skills (self)	0.28	0.32	−0.22	−0.42^**^	0.44^***^	−0.22	−0.25	−0.25	—						
10. Social skills (partner)	0.08	0.28	0.05	−0.09	0.24	−0.09	−0.24	−0.22	0.58^**^	—					
11. Similarity	−0.08	0.13	−0.09	−0.13	0.22	−0.13	−0.11	−0.27	0.09	0.27	—				
12. Affiliation	−0.07	0.13	−0.20	−0.17	0.35	−0.13	−0.22	−0.26	0.10	0.23	0.68^***^	**—**			
13. Factor 1 (similarity)	0.06	−0.06	−0.27	−0.20	0.34	−0.08	−0.24	−0.27	0.13	0.14	0.64^***^	0.86^***^	**—**		
14. Factor 2 (positive qualities)	−0.23	0.24	−0.02	−0.16	0.27	−0.14	−0.10	−0.21	0.01	0.19	0.56^***^	0.75^***^	0.46^***^	—	
15. Factor 3 (comfort)	−0.01	0.24	−0.13	−0.14	0.23	−0.13	−0.19	−0.27	0.17	0.37	0.56^***^	0.74^***^	0.50^***^	0.55^***^	—

*The asterisk(s) following the coefficients indicate the degree of strength of evidence in favor of the existence of a correlation between the variables based on Bayes Factor (BF): BF_10_: 3–10 (moderate), BF_10_: 10–30 (strong), BF_10_: > 100 (extreme). Credible correlations are presented in bold with asterisks referring to the strength of evidence*.

* *BF_10_ > 10*.

** *BF_10_ > 30*.

*** *BF_10_ > 100*.

The following results are presented by research question.

### Do Distinct PL Patterns Emerge Across Dyads?

**Figure 1:**
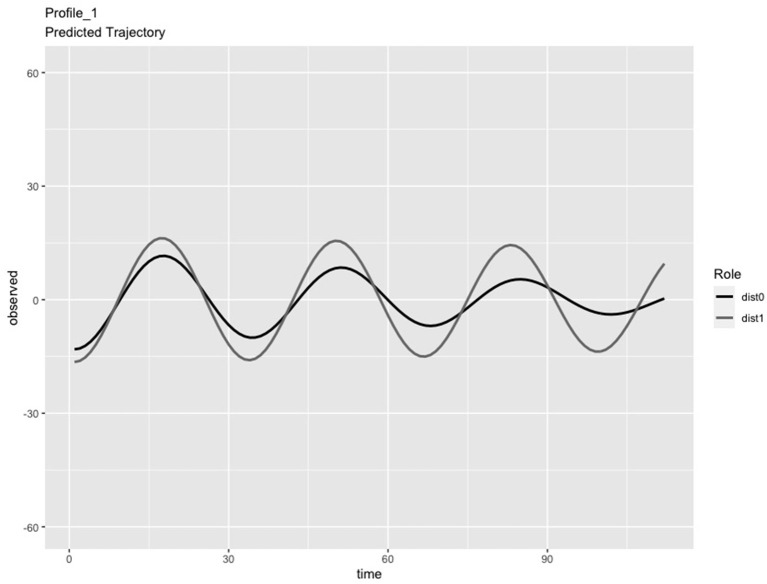
Profile 1, labeled as Simple-Slow, was characterized by slow frequency, in-phase synchronization that showed one person's signal damping over time (n = 27 dyads).

**Figure 2:**
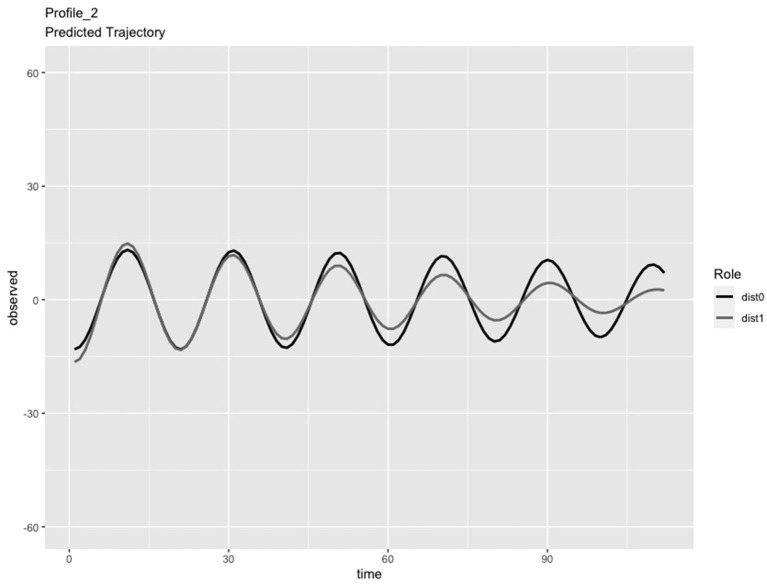
Profile 2, labeled as Simple-Fast, was characterized by a fast frequency, in-phase synchronization pattern that also showed one person's signal damping over time (n = 17 dyads).

**Figure 3:**
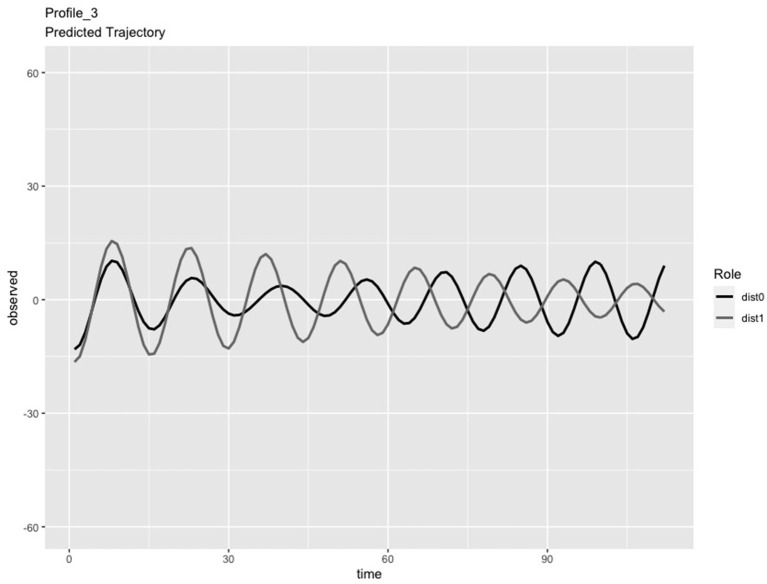
Profile 3, labeled as the Complex Profile, characterized by a fast frequency, shifting to anti-phase pattern (n = 21 dyads).

In the LPA step of the analysis, we compared solutions consisting of 2 and 3 profiles. We chose the 3-profile solution because: (a) the predicted IBI trajectories for the 3-profile solution were visually distinct (see Figures 1–3), whereas the trajectories in the 2-profile solution were very similar to each other; and (b) membership in one profile of the 2-profile solution did not contain enough dyads (i.e., <10% of 65 dyads) and (c) the dynamics depicted in the 3-profile solution were similar to patterns found using the same method in a prior study (Li X. et al., 2020). To visualize the PL patterns over the course of the task, we plotted the predicted trajectories. As seen in Figure 1, Profile 1 was characterized by slow frequency, in-phase synchronization that showed one person's signal damping over time (n = 27 dyads). As seen in Figure 2, Profile 2 was characterized by a fast frequency, in-phase synchronization pattern that showed one person's signal damping over time (n = 17 dyads). As seen in Figure 3, Profile 3 was characterized by a fast frequency, shifting to anti-phase pattern (n = 21 dyads). Across all profiles, one partner's signal appears to damp over time with Profile 3 showing less stability over time compared to Profile 1 and 2. Thus, we labeled Profile 1 as “Simple-Slow,” Profile 2 as “Simple-Fast,” and labeled Profile 3 as “Complex.” Profile membership was written as a variable in the data frame and used in subsequent analyses.

### Do PL Patterns Predict Affiliation?

Results suggested that PL profile was not a likely predictor of any affiliation-related variables. Specifically, the 90% HDI for all relevant parameters included zero.

### Do PL Patterns Predict Affiliation Differently Across Conditions?

Results suggested that PL profile and condition did not interact to predict any affiliation-related variables. Specifically, the 90% HDI for all relevant parameters included zero.

### Are PL Patterns Predicted by Condition?

Results suggested that condition did not predict the probability of being in different PL profiles. Specifically, the 90% HDI for all relevant parameters included zero.

### Are PL Patterns Predicted by Individual Differences or Affiliation?

As seen in Table 5, the results showed that both averages and differences in individual variables differentially predicted PL patterns. More specifically, greater differences in self-rated social skills, ratings of affiliation, perception of partner's positive qualities, and comfort during interaction were associated with a greater probability of being in Profile 1 (Simple-Slow) than Profile 3 (Complex). Higher average levels of avoidant attachment predicted a greater probability of being in Profile 1 (Simple-Slow) than Profile 2 (Simple-Fast). Higher mean attachment security predicted a greater probability of being in Profile 2 (Simple-Fast) than Profile 3 (Complex). In summary, Profile 1 (Simple-Slow) was associated with partner's reporting mismatched levels of positive social characteristics and higher levels of negative characteristics, while Profile 2 (Simple-Fast) was associated with higher security. Finally, Profile 3 (Complex) was associated with more concordant partner reports of positive characteristics and lower reports of negative characteristics, but also lower security.

**Table 5:** Individual-level variables predicting PL pattern.

		**Profile 1 (simple-slow) vs. profile 2 (simple-fast)**		**Profile 1 (simple-slow) vs. profile 3 (complex)**	
**Outcome**	**Predictor**	**Average**	**Difference**	**Average**	**Difference**
PL pattern	Interaction anxiousness	[−1.74, 0.03]	[−0.86, 0.31]	[−1.56, 0.10]	[−0.53, 0.56]
	Secure attachment	[−0.28, 1.66]	[−0.54, 0.58]	[−1.46, 0.52]	[−0.69, 0.40]
	Avoidant attachment	**[−3.06**, **−0.25]**	[−0.82, 0.61]	[−1.97, 0.48]	[−0.28, 1.06]
	Anxious attachment	[−1.58, 0.04]	[−0.57, 0.36]	[−0.45, 1.21]	[−0.20, 0.69]
	Social anhedonia	[−1.13, 0.75]	[−0.18, 0.86]	[−1.53, 0.31]	[−0.34, 0.64]
	Social skills (self)	[−0.61, 0.85]	[−0.54, 0.38]	[−1.11, 0.27]	**[−0.90**, **−0.05]**
	Social skills (partner)	[−0.60, 0.88]	[−0.39, 0.50]	[−0.63, 0.76]	[−0.52, 0.31]
	Similarity	[−0.47, 0.94]	[−0.37, 0.61]	[−0.61, 0.74]	[−0.19, 0.74]
	Affiliation	[−0.79, 0.60]	[−1.00, 0.32]	[−0.80, 0.56]	**[−1.61**, **−0.26]**
	Factor 1 (similarity)	[−0.64, 0.66]	[−0.39, 0.49]	[−0.97, 0.29]	[−0.48, 0.29]
	Factor 2 (positive qualities)	[−0.89, 0.62]	[−1.22, 0.18]	[−0.44, 0.93]	**[−1.40**, **−0.10]**
	Factor 3 (comfort)	[−0.67, 0.65]	[−0.68, 0.24]	[−0.70, 0.68]	**[−1.34**, **−0.34]**

*90% HDIs are presented in brackets. Given that there was only one credible finding from Profile 2 compared to Profile 3, the column comparing Profile 2 to Profile 3 was omitted from this table. As mean attachment security increased, the probability of being in Profile 2 (Simple-Fast) increased compared to Profile 3 (Complex) [−2.24, −0.04]. Credible predictors are presented in bold*.

### Are PL Patterns Predicted by Individual Differences or Affiliation Differently Across Conditions?

As seen in Table 6, results showed that individual differences interacted with condition to differentially predict PL patterns. More specifically, Profile 1 (Simple-Slow) was most likely in the Competitive/No-Talking condition at: (a) higher mean levels of attachment avoidance and attachment anxiety, (b) lower differences in attachment avoidance and social anhedonia, and (c) higher differences of attachment security and self-rated social skills. Profile 3 (Complex) was most likely in the Collaborative/Talking condition at lower mean comfort level during the interaction.

**Table 6:** Interaction of individual-level variables and condition predicting PL patterns.

**Most likely profile**	**Condition**	**Individual-level variable**	**HDI**
**Profile 1:** **simple-slow** (vs. Profile 2)	Competitive/ no talking	↑ Mean attachment avoidance	[−3.74, −0.33]
		↑ Mean attachment anxiety	[−2.18, −0.43]
		↓ Difference in attachment avoidance	[0.57, 2.45]
		↓ Difference in social anhedonia	[0.11, 1.19]
		↑ Difference in attachment security	[−1.15, −0.02]
		↑ Difference in self-rated social skills	[−0.97, −0.04]
**Profile 3:** **complex** (vs. profile 1)	Collaborative/ talking	↓ Mean comfort	[0.11, 1.70]

*90% HDIs are presented in brackets. ↑ Represents higher, ↓ Represents lower*.

## Discussion

### Summary and Key Findings

We examined physiological linkage (PL) using data from 65 same-sex, same ethnicity stranger dyads. Using the *rties* package (Butler and Barnard, 2019), we modeled different oscillatory patterns of interdependence between partner's interbeat intervals. We found three patterns of PL characterized by differences in frequency of oscillation, phase, damping, and amplification. More specifically, we found two profiles that showed in-phase synchronization with slow frequency (Profile 1) and fast frequency (Profile 2). The remaining profile showed fast frequency with drifting anti-phase synchronization (Profile 3). The profiles were labeled Simple-Slow, Simple-Fast, and Complex, respectively. We explored these PL patterns as predictors of affiliation and as an outcome of the interaction between individual differences and experimental condition. Our exploratory analyses showed that PL patterns *per-se* or their interaction with condition did not predict affiliation. Furthermore, experimental condition was not a likely predictor of PL patterns.

Although we did not find support for the bidirectional relationship between PL and affiliation, we found that individual differences and affiliation predicted PL patterns. Collectively, we found that greater discrepancies between partners on ratings of one's own social skills pre-task and ratings of the interaction post-task (e.g., affiliation, partner's positive qualities, and comfort during the interaction) predicted a higher likelihood of belonging to the Simple-Slow Profile compared to the Complex Profile. Additionally, we found that membership in the Simple-Slow Profile compared to the Simple-Fast Profile was more likely at higher average levels of avoidant attachment. Regarding the interaction of individual differences and condition to predict PL patterns, we found that the Simple-Slow Profile was most likely in the Competitive/No Talking condition when there were greater differences in attachment security and self-rated social skills as well as greater mean levels of avoidant and anxious attachment. Finally, the Complex Profile was most likely during the Collaborative/Talking condition when there were lower mean comfort levels during the interaction.

Taken together, these results highlight the importance of including individual differences and context when examining PL, including the consideration of both the dyad average and difference on individual-level variables. Interestingly, both global (e.g., attachment style) and specific interpersonal variables (e.g., feelings about an upcoming interaction and ratings of it after) predicted PL patterns. Furthermore, the dyad average attachment style results were between the two simple profiles (Profile 1 and Profile 2), while the within dyad differences relating to the post-task measures were between a simple (Profile 1) and complex profile (Profile 3). These findings suggest that mismatch on individual-level variables related to the interaction could be influencing PL during the interaction. Regarding context, the simple profiles were more likely at higher levels of traits that have been shown to make people less likely to engage with others (e.g., attachment anxiety and avoidance), which the competitive context may have exacerbated. When one disengages with their social environment, there are fewer opportunities for external perturbations to one's physiological baseline, which could explain the simple profiles. The Complex Profile was seen in the Collaborative/Talking Condition at lower average comfort levels during the interaction. Given that there are more opportunities for external perturbation in this condition (e.g., turn-taking during a conversation, higher-order cooperation, and the presence of a partner who makes one feel uncomfortable), the Complex profile could be a result of coregulation during the task and/or of overall interaction quality.

### Limitations and Future Directions

We have several limitations to consider. First, we could not distinguish signals from the sympathetic and parasympathetic nervous systems, given that interbeat interval reflects joint action of the SNS and PNS. This limitation could explain why our PL patterns were not predictive of affiliation as seen in Danyluck and Page-Gould (2019) who modeled linkage in each branch separately using the same dataset. Second, given that participants were matched on sex and ethnicity, we utilized an arbitrary distinguishing variable (i.e., 0 vs. 1) for the dyads. Therefore, we cannot say which partner was damping over time in each of the profiles. However, the *rties* package allows one to distinguish individuals in a dyad based on any characteristic of interest. Thus, the modeling capabilities of the package could be used to examine a variety of dyad types and make conclusions about individual dynamics. Third, since we used cross-sectional measures, it is difficult to untangle the reciprocal relationship between PL and affiliation. Future studies should consider designs that test the reciprocity of higher-order social processes and PL and use sample sizes large enough to validate the cluster stability in the LPA step of the analysis. Despite these limitations, this study highlights the importance of (a) capturing dynamic patterns of PL and (b) considering individual differences and context when investigating PL. Although speculative, the simple-slow pattern we observed could indicate social disengagement, resulting from pre-existing intrapersonal traits that promote antisociality, partner mismatch on these traits, and/or the demands of the context. Furthermore, these results could generalize to other physiological measures (e.g., respiratory sinus arrhythmia and skin conductance), contexts, and dyad types. More specifically, this work could inform studies examining PL among individuals who have just met and plan to interact in the future (e.g., therapist-client, teacher-student, and teammate-teammate). Future work will need to focus on uncovering the combinations of individual differences and contexts that generate such patterns.

## Data Availability Statement

The data that support the findings of this study are available on request from the corresponding author (SB). The data are not publicly available because consent to make these data public was not obtained from participants a priori and thus we do not have ethics approval to post it online. Relevant code is hosted at https://osf.io/x7baf/.

## Ethics Statement

The studies involving human participants were reviewed and approved by University of Toronto Institutional Review Board. The patients/participants provided their written informed consent to participate in this study.

## Author Contributions

SB conducted the analyses and wrote the manuscript. AK, EP-G, and EB helped with manuscript revisions. CD and EB supervised SB during the process of data analysis, writing, and revisions and helped with editing manuscript drafts. All authors contributed to the article and approved the submitted version.

## Funding

This work was supported by Social Sciences and Humanities Research Council of Canada (SSHRC) Operating grants 435130767 and 410101498 (to EP-G) and a SSHRC Doctoral Fellowship (to CD).

## Conflict of Interest

The authors declare that the research was conducted in the absence of any commercial or financial relationships that could be construed as a potential conflict of interest.

## Publisher's Note

All claims expressed in this article are solely those of the authors and do not necessarily represent those of their affiliated organizations, or those of the publisher, the editors and the reviewers. Any product that may be evaluated in this article, or claim that may be made by its manufacturer, is not guaranteed or endorsed by the publisher.
